# Clinical and Microbiological Insights Into Burkholderia Infections: A Retrospective Study From a Tertiary Care Hospital

**DOI:** 10.7759/cureus.76742

**Published:** 2025-01-01

**Authors:** Shubhransu Patro, Vibha Sharma, Arushi Choudhary, Yallambhotla Varuneil, Basanti Kumari Pathi, Sidharth S Pattnaik

**Affiliations:** 1 General Medicine, Kalinga Institute of Medical Sciences, Bhubaneswar, IND; 2 Microbiology, Kalinga Institute of Medical Sciences, Bhubaneswar, IND

**Keywords:** antibiotic resistance, burkholderia cepacia, burkholderia pseudomallei, inflammatory markers, tertiary care hospital

## Abstract

Background

Opportunistic pathogens such as *Burkholderia cepacia *and *B. pseudomallei *within the genus *Burkholderia* are significant causes of morbidity and mortality, especially in immunocompromised individuals. Despite their clinical importance, these infections are often underreported in resource-poor settings due to diagnostic challenges. This study investigates the prevalence, clinical profiles, and antibiotic resistance patterns seen in *Burkholderia* infections in a tertiary care hospital in India.

Methods

This retrospective study analyzed 56 hospitalized patients with *Burkholderia* infections diagnosed between June 2022 and June 2024. Positive cultures were identified using biochemical and morphological criteria. Demographic, clinical, and laboratory data, as well as antibiotic susceptibility profiles, were reviewed. Statistical analysis included descriptive measures and comparisons between *B. cepacia* and *B. pseudomallei*.

Results

The study included 56 patients, predominantly male (66.10%), with a mean age of 50.10 years. Comorbidities such as diabetes (39.30%) and hypertension (35.70%) were common. Blood cultures were the most frequent sample type, yielding positive results, with *B. cepacia* isolated in 67.60% of cases and *B. pseudomallei *in 32.40%. Patients with *B. pseudomallei *exhibited a stronger systemic inflammatory response, reflected by significantly elevated procalcitonin levels (p = 0.035) compared to *B. cepacia*. While C-reactive protein levels were also higher in *B. pseudomallei*, the difference was not statistically significant (p = 0.066). Both species demonstrated sensitivity to carbapenems and beta-lactam/beta-lactamase inhibitors, but notable resistance to aminoglycosides and cephalosporins was observed. *B. pseudomallei *showed high resistance to amikacin (41.20%) and cefepime (41.20%) while *B. cepacia* exhibited resistance to piperacillin/tazobactam (30.8%) and aztreonam (30.80%).

Conclusion

This study highlights distinct clinical and microbiological characteristics of *B. cepacia* and *B. pseudomallei* with distinct inflammatory markers and resistance patterns that underscore the need for precise diagnostic tools and tailored antibiotic therapy. Carbapenems and beta-lactam/beta-lactamase inhibitors remain effective treatment options, but emerging resistance necessitates effective antimicrobial stewardship for combating antimicrobial resistance and ensuring optimal patient outcomes. Further multicentric studies are essential to validate these findings and optimize management strategies for *Burkholderia* infections.

## Introduction

The genus *Burkholderia*, part of the *Burkholderiaceae* family, comprises a diverse group of non-fermenting Gram-negative bacteria (NFGNB) known for their remarkable metabolic adaptability and environmental resilience [1]. Initially described by Walter Burkholder in 1942 as a phytopathogenic organism affecting crops, *Burkholderia* species have since been recognized as both beneficial and pathogenic [2]. While certain strains within this genus contribute to biotechnological and agricultural applications such as bioremediation and biocontrol, several *Burkholderia* species have emerged as significant opportunistic pathogens, particularly affecting immunocompromised individuals [3].

Among the pathogenic species, *Burkholderia pseudomallei* and the *B. cepacia *complex (BCC) stand out due to their clinical significance.* B. pseudomallei*, the causative agent of melioidosis, is associated with severe and often fatal infections in tropical regions, including Southeast Asia and Northern Australia, and exhibits significant resistance to various antibiotics. BCC, which comprises at least 17 species, presents a particular threat to cystic fibrosis (CF) patients due to its role as a nosocomial pathogen, leading to high morbidity and mortality rates in this vulnerable population [3,4]. In South Asia, reports suggest that melioidosis might account for 44% of the global disease burden, with India suspected to have a substantial yet underrated prevalence [5].

Despite the pathogenic potential of *Burkholderia* species, their clinical importance is often underestimated in developing countries, where diagnostic challenges contribute to underreporting and delayed treatment. Many clinical laboratories may misidentify *Burkholderia* isolates as *Pseudomonas* species or dismiss them as contaminants. Further complicating the issue, advanced diagnostic tools such as VITEK® 2 Compact (bioMerieux, Marcy-l'Étoile, France), MALDI-TOF mass spectrometry, and 16S ribosomal RNA sequencing require specialized expertise and are not widely accessible, especially in resource-limited settings [6]. As a result, the incidence of *Burkholderia* infections is likely underreported, leading to unacknowledged pervasiveness and inadequate management of these infections.

This study aims to provide comprehensive insights into the epidemiology and clinical outcomes of *Burkholderia* species infections in an area that has received limited attention in existing literature. Additionally, the study seeks to determine the antibiotic sensitivity pattern, which is crucial for developing effective antimicrobial stewardship in the face of growing antimicrobial resistance.

By retrospectively analyzing cases, we aim to improve the understanding and address the knowledge gap of the clinical and microbiological characteristics of *Burkholderia* infections, with an emphasis on enhancing diagnostic accuracy and treatment strategies.

## Materials and methods

Study design and setting

This retrospective observational cross-sectional study was conducted at the Kalinga Institute of Medical Sciences, Bhubaneshwar, over a two-year period (June 15, 2022 to June 15, 2024). The study aimed to evaluate the prevalence, predisposing conditions, clinical presentations, and outcomes of hospitalized patients diagnosed with infections caused by *Burkholderia* species, as well as assess the antibiotic sensitivity patterns of these isolates.

Inclusion and exclusion criteria

Patients aged 18 years or above with positive cultures for *Burkholderia* species were included in the study. Only patients admitted to the hospital were considered for analysis to ensure complete clinical records and culture data. Outpatients and those with incomplete records were excluded from the study.

Data collection and processing

Specimen samples received from patients were subjected to Gram staining and subsequently inoculated onto sheep blood agar (5%, Thermo Fisher Scientific, Waltham, Massachusetts, USA) and MacConkey agar plates (Thermo Fisher Scientific, Waltham, Massachusetts, USA). The plates were incubated aerobically at 37°C for a minimum of five days. Identification of *Burkholderia* species was based on colony morphology, growth characteristics, and biochemical reactions. NFGNB were further classified as *Burkholderia* species by evaluating oxidative utilization of glucose, lactose, mannitol, and maltose, liquefaction of gelatin, nitrate reduction, and arginine hydrolysis. Demographic data and clinical records of patients with *Burkholderia* infections were retrieved to assess clinical features, underlying risk factors (such as age, gender, chronic disease history, and immunosuppressive conditions), and laboratory parameters at the time of sample collection. This analysis also included antibiotic susceptibility patterns based on standard microbiological protocols to determine effective treatment options.

All cases meeting the inclusion criteria during the study period were analyzed following a consecutive sampling approach.

Statistical analysis

Descriptive statistics were computed using “R” version 4.3.2 (R Foundation, Vienna, Austria). Categorical variables, including demographic data, clinical features, and antibiotic susceptibility patterns, were summarized using frequencies and percentages (Chi-square for categorical variables and t-test for continuous variables). The lab parameters were compared using Mann-Whitney U test and t-test.

This study was performed in accordance with the principles of the Declaration of Helsinki, with ethical approval obtained from the Institutional Ethics Committee of Kalinga Institute of Medical Sciences (KIIT/KIMS/IEC/1887/2024) prior to the study's commencement. Informed consent was waived by the Institutional Ethics Committee due to the retrospective nature of the study and anonymized data analysis.

## Results

Demographic analysis

**Figure 1:**
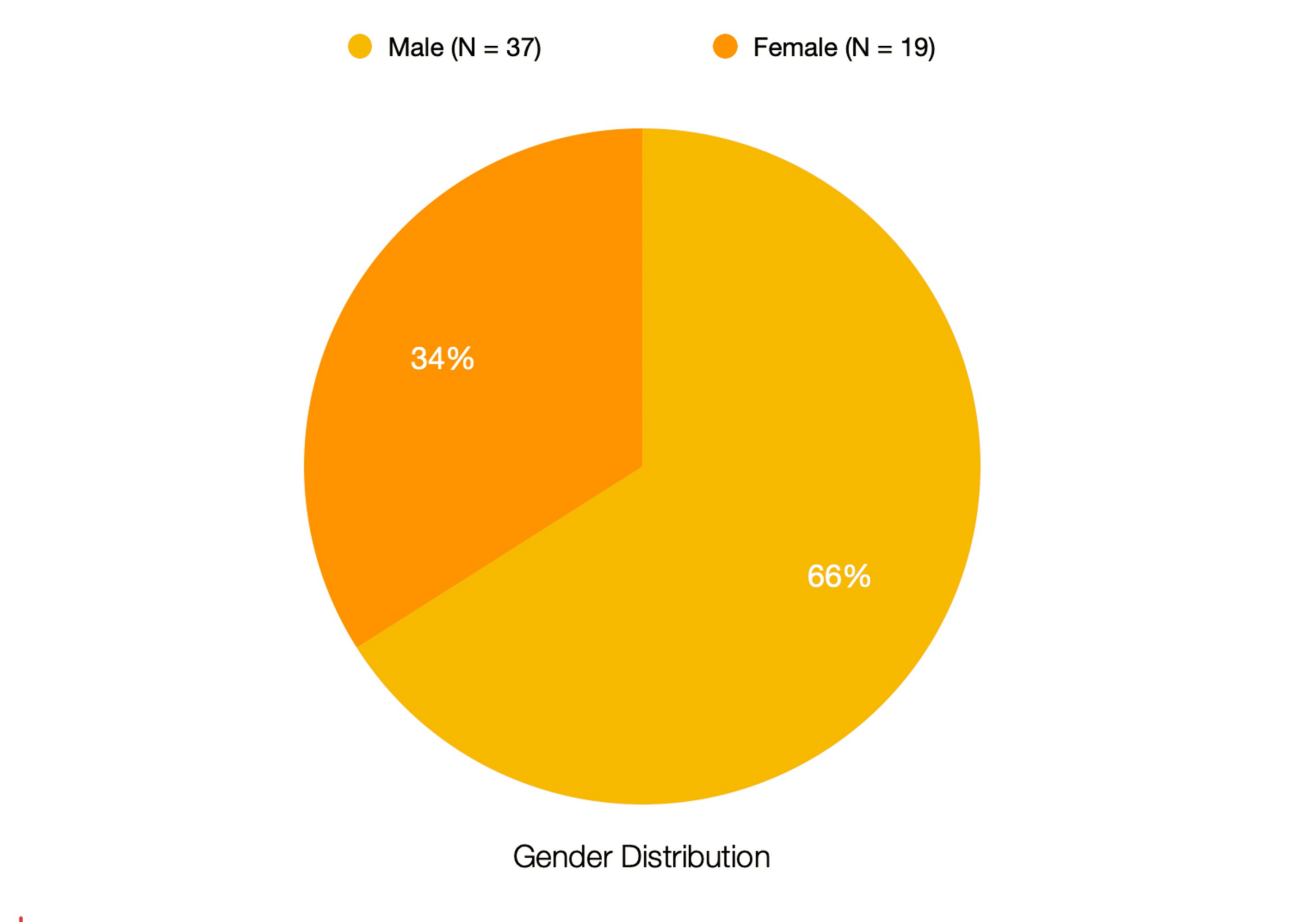
Overall gender distribution

The demographic characteristics of the patient cohort are summarized in Table 1. A total of 56 patients were analyzed, with a male predominance (66.1%) as shown in Figure 1. The age distribution ranged from 6 months to 81 years, with the majority (46.4%) being between 40 and 60 years old. The prevalence of hypertension was noted in 25% of patients, while 39.3% had diabetes. Smoking was reported in 19.64% and alcohol consumption was noted in 21.42% of cases.

**Table 1:** Patient demographics The demographic data have been expressed as frequency (proportion).

Demographic Variable	Category	N	Percentage (%)
Total Patients		56	100
Gender	Male	37	66.10
	Female	19	33.90
Age Group	0-20	2	3.60
	21-30	4	7.10
	31-40	8	14.30
	41-50	11	19.60
	51-60	13	23.20
	61-70	11	19.60
	71-80	7	12.50
Hypertension	Yes	14	25
	No	42	75
Diabetes	Yes	22	39.30
	No	34	60.70
Smoking	Yes	11	19.64
	No	45	80.35
Alcohol	Yes	12	21.42
	No	44	78.57

**Figure 2:**
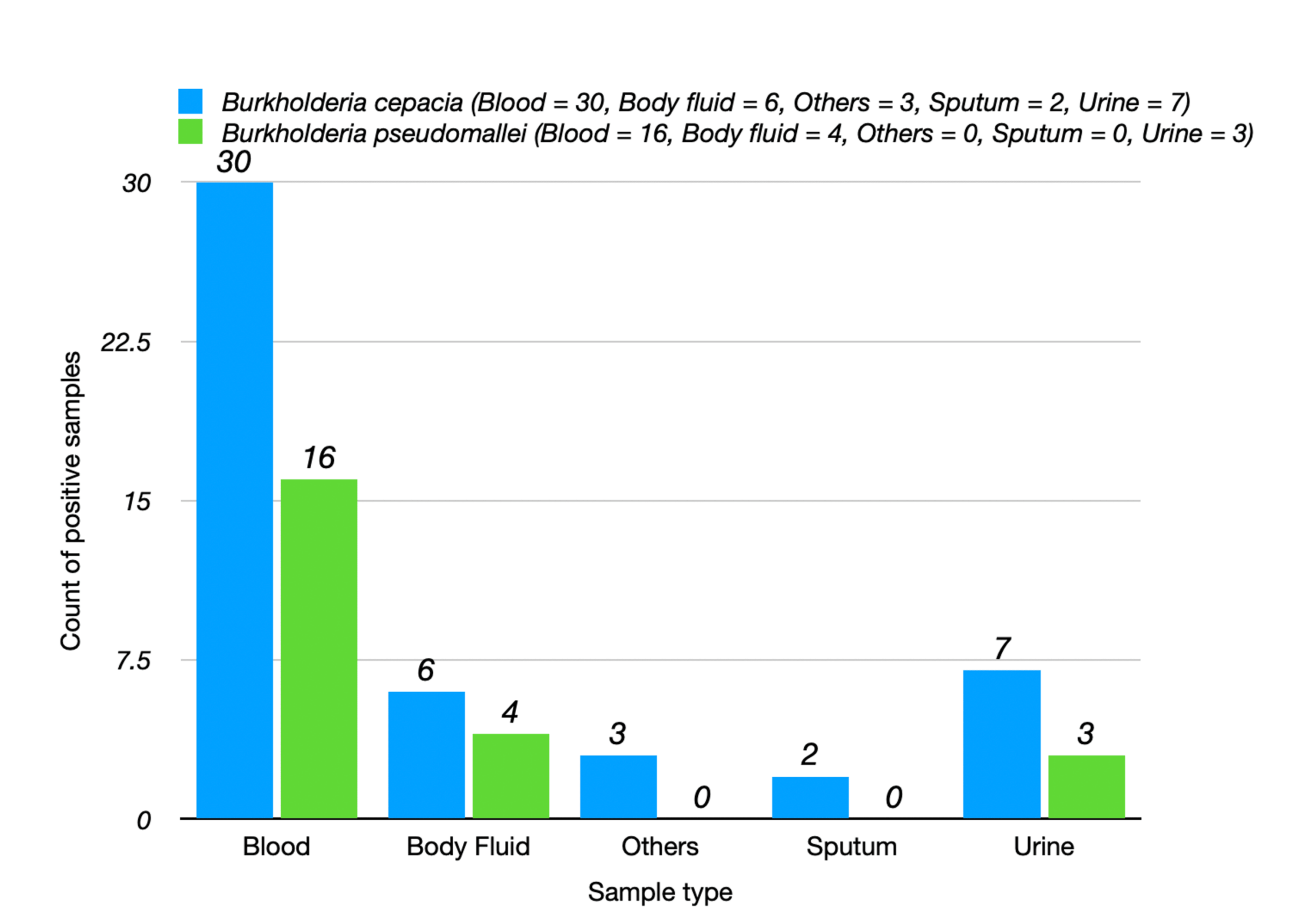
Distribution of Burkholderia by sample type

A total of 56 patients were included in this study, with samples collected for culture analysis across various types, including blood, urine, sputum, others, and body fluids as shown in Figure 2. The positive culture results revealed the presence of two species of *Burkholderia: B. cepacia* and *B. pseudomallei*​​​​​​*.*

The proportion of positive samples for *B. cepacia* represented approximately 67.61% of the total samples, while *B. pseudomallei* accounted for about 32.39% as shown in Table 2. The ratio of positive samples for *B. cepacia* to *B. pseudomallei* was calculated to be approximately 2.09, indicating that *B. cepacia* was more prevalent than *B. pseudomallei *in the sampled population.

**Table 2:** Summary table of positive samples with percentages Total 56 patients have been included with 71 samples. The percentages of total positive samples have been calculated based on total 71 samples.

Sample Type	*Burkholderia cepacia* (N)	*B. pseudomallei* (N)
Blood	30	16
Urine	7	3
Sputum	2	0
Others	3	0
Body Fluid	6	4
Total Positive Samples	48	23
Percentage	67.61%	32.39%

Demographics

The study included a total of 56 patients, with 39 diagnosed with *B. cepacia* and 17 with *B. pseudomallei*.

**Figure 3:**
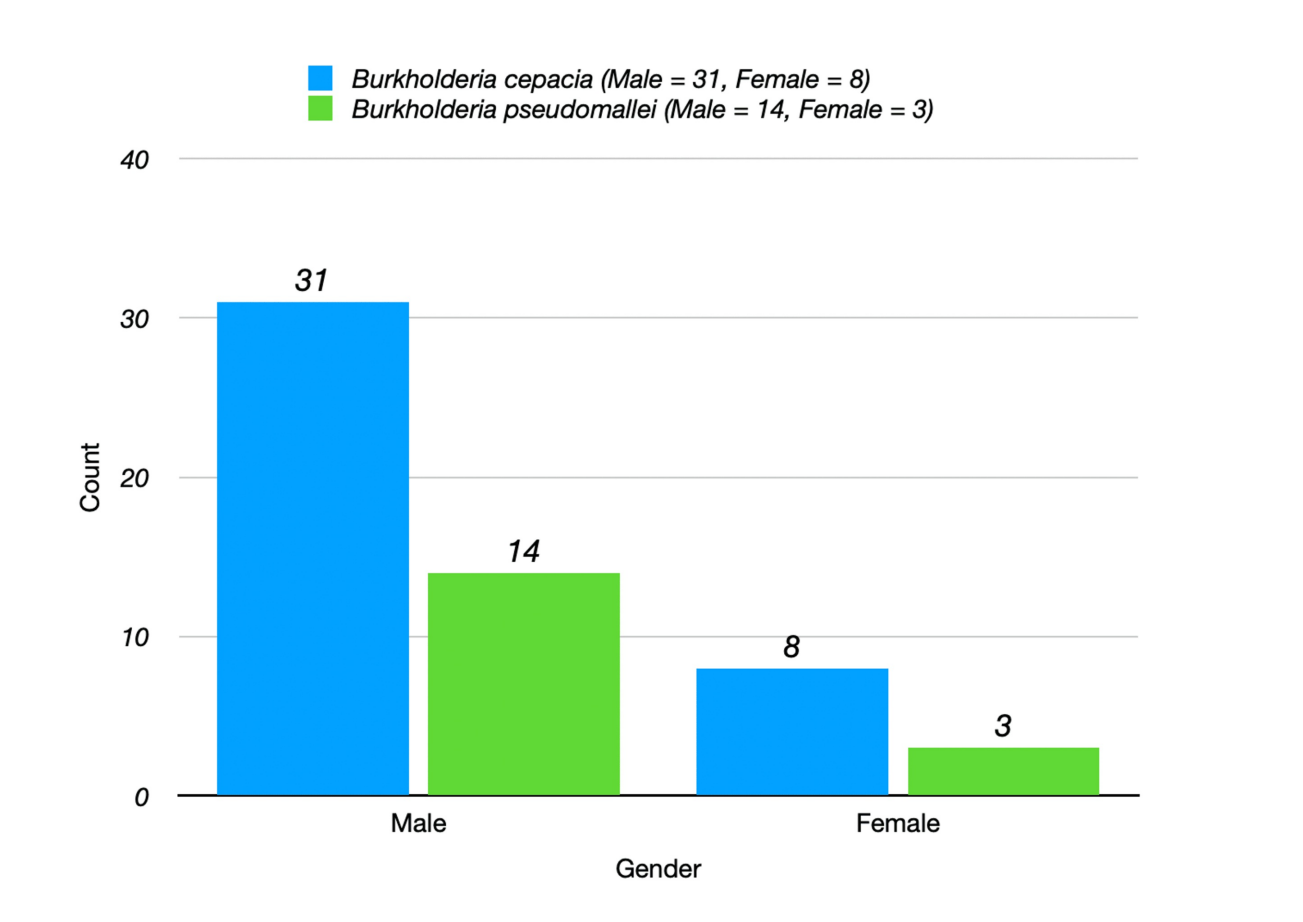
Gender distribution

**Figure 4:**
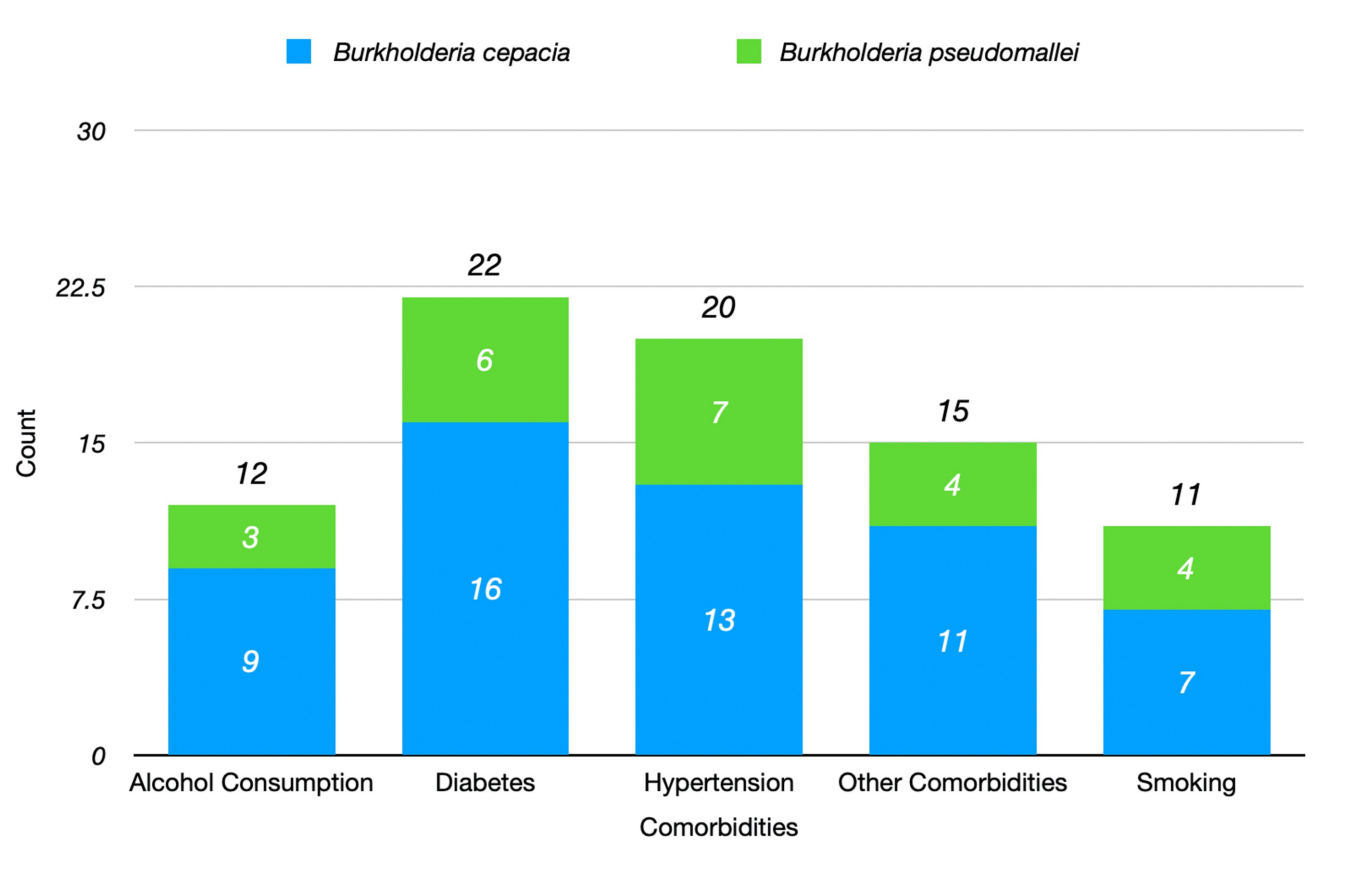
Comorbidities by species

The average age of patients with *B. cepacia* was 49.50 years, while those with *B. Psudomallei* had a mean age of 52.10 years. A higher male prevalence was observed in both groups, with 31 males in the *B. cepacia* group and 14 males in the* B. pseudomallei* group as shown in Figure 3. The prevalence of associated comorbidities varied between both the species as shown in Figure 4. Among the 39 *B. cepacia* patients, 33.33% had hypertension compared to 41.18% in the *B. pseudomallei* group. The prevalence of diabetes was slightly higher in *B. cepacia* (41.03%) than in *B. pseudomallei* (35.29%). While hypertension and diabetes were slightly more prevalent in *B. pseudomallei* patients, these differences were not statistically significant, suggesting comparable risk profiles between the two groups. A total of 28.21% of *B. cepacia* patients reported other comorbidities compared to 23.53% of *B. pseudomallei *patients. Smoking was reported in 17.95% of *B. cepacia* patients and 23.53% of *B. pseudomallei* patients. Alcohol consumption was noted in 23.08% of *B. cepacia* patients compared to 17.65% of *B. pseudomallei* patients.

The data in Table 3 highlight differences in clinical parameters between patients with *B. cepacia* and *B. pseudomallei.* While hypertension and diabetes were common across both groups, *B. pseudomallei *patients exhibited a slightly higher prevalence of these conditions. Understanding these clinical characteristics can aid in the management and treatment strategies for patients with infections caused by these organisms.

**Table 3:** Comparison of two species by lab parameters (Mann-Whitney U test/t-test) The continuous variables have been expressed as mean and SD. CRP, C-reactive protein; ESR, erythrocyte sedimentation rate; MPV, mean platelet volume; PDW, platelet distribution width; PLT, platelet count; SD, standard deviation; WBC, white blood cell

Parameter	Mean Value (in *Burkholderia pseudomallei*)	Standard Deviation (*B. pseudomallei*)	Mean Value (in *B. cepacia*)	Standard Deviation (*B. cepacia*)	p-value
ESR	60.47	27.79	55.69	25.29	0.54
CRP	197.23	98.79	145.83	98.62	0.06
Procalcitonin	44.89	40.72	31.78	57.29	0.03
MPV	9.98	1.15	10.24	1.22	0.44
PDW	12.59	3.34	13.44	2.30	0.72
PLT	189.29	109.65	180.56	103.96	0.88
WBC	12.27	6.62	14.59	10.02	0.55
Lymphocyte	10.47	7.18	10.81	7.36	0.86
Neutrophil	83.66	7.19	80.39	16.18	0.60

**Figure 5:**
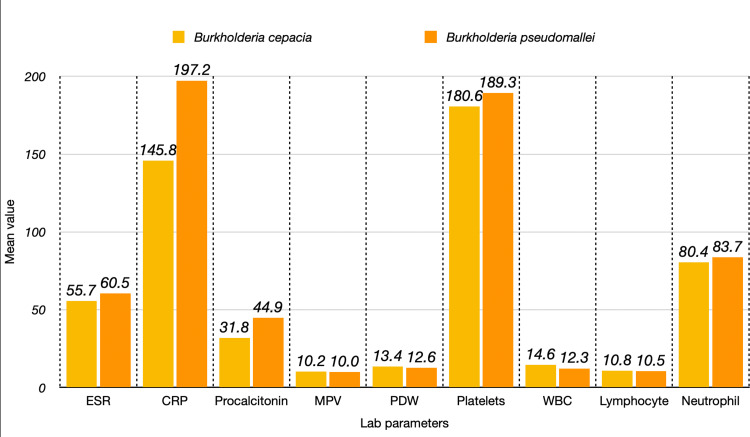
Comparison of clinical parameters between two Burkholderia species CRP, C-reactive protein; ESR, erythrocyte sedimentation rate; MPV, mean platelet volume; PDW, platelet distribution width; PLT, platelet count; WBC, white blood cell

The analysis of laboratory parameters between *B. pseudomallei* and *B. cepacia* reveals several interesting insights regarding the inflammatory responses associated with these infections as shown in Table 4 and Figure 5.

**Table 4:** Comparative analysis of demographic and clinical parameters by species The categorical and continuous data have been expressed as frequency (proportion) and mean, respectively.

Parameter	Burkholderia cepacia	B. pseudomallei	Total Patients
Total Patients (N)	39	17	56
Mean Age (Years)	49.50	52.10	50.10
Gender Distribution (N)			
Male	31	14	45
Female	8	3	11
Hypertension (N[%])			
Yes	13 (33.33%)	7 (41.18%)	20 (35.71%)
No	26 (66.67%)	10 (58.82%)	36 (64.29%)
Diabetes (N[%])			
Yes	16 (41.03%)	6 (35.29%)	22 (39.29%)
No	23 (58.97%)	11 (64.71%)	34 (60.71%)
Other Comorbidities (N[%])			
Yes	11 (28.21%)	4 (23.53%)	15 (26.79%)
No	28 (71.79%)	13 (76.47%)	41 (73.21%)
Smoking (N[%])			
Yes	7 (17.95%)	4 (23.53%)	11 (19.64%)
No	32 (82.05%)	13 (76.47%)	45 (80.36%)
Alcohol Consumption (N[%])			
Yes	9 (23.08%)	3 (17.65%)	12 (21.43%)
No	30 (76.92%)	14 (82.35%)	44 (78.57%)

The erythrocyte sedimentation rate (ESR) was found to have a mean of 60.47 mm/h (standard deviation [SD]: 27.79) in the *B. pseudomallei *group compared to a mean of 55.69 mm/h (SD: 25.29) in the *B. cepacia* group. The p-value of 0.548 indicates that there is no statistically significant difference in ESR between the two species. This suggests that the inflammatory response, as reflected by ESR, is comparable in patients infected with either organism.

C-reactive protein (CRP) levels showed a mean of 197.23 mg/L (SD: 98.79) for *B. pseudomallei *group, whereas the *B. cepacia* group had a mean of 145.83 mg/L (SD: 98.62). The p-value of 0.066, while not meeting the conventional significance threshold of 0.05, approaches it. This indicates a trend toward a stronger inflammatory response in *B. pseudomallei *infections, as evidenced by the higher mean CRP levels. Procalcitonin levels exhibited a notable difference between the two groups, with a mean of 44.89 ng/mL (SD: 40.72) in *B. pseudomallei* and a mean of 31.78 ng/mL (SD: 57.29) in *B. cepacia*. The p-value of 0.035 indicates a statistically significant difference, suggesting that patients with *B. pseudomallei* infection have a more pronounced systemic inflammatory response or potentially greater infection severity than those with *B. cepacia*. The mean platelet volume (MPV) was similar between the two species, with *B. pseudomallei* showing a mean of 9.98 fL (SD: 1.15) and *B. cepacia* showing a mean of 10.24 fL (SD: 1.22). The p-value of 0.44 suggests no significant difference in MPV, indicating that platelet activity does not vary notably between infections caused by these two organisms. For platelet distribution width (PDW), *B. pseudomallei* had a mean of 12.59% (SD: 3.34), while* B. cepacia* had a mean of 13.44% (SD: 2.30). The p-value of 0.72 indicates that there is no significant difference in PDW between the two species, implying consistent variability in platelet sizes across both infections. In terms of platelet count (PLT), the mean for *B. pseudomallei* was 189.29 × 109/L (SD: 109.65), and for *B. cepacia,* it was 180.56 × 109/L (SD: 103.96). The high p-value of 0.887 indicates no significant difference in PLTs between the two groups, suggesting a similar platelet response in both infections. WBC counts also did not demonstrate a significant difference, with *B. pseudomallei *having a mean of 12.27 × 109/L (SD: 6.62) and *B. cepacia* showing a mean of 14.59 × 109/L (SD: 10.02). The p-value of 0.55 suggests that the immune response is comparable for both types of infections. The analysis of lymphocyte counts revealed a mean of 10.47 × 109/L (SD: 7.18) for* B. pseudomallei* and a mean of 10.81 × 109/L (SD: 7.36) for *B. cepacia*. With a p-value of 0.86, there is no significant difference, indicating a similar lymphocytic response in both infections.

Finally, neutrophil counts were also similar across the two species, with *B. pseudomallei* showing a mean of 83.66 × 109/L (SD: 7.19) and *B. cepacia *having a mean of 80.39 × 109/L (SD: 16.18). The p-value of 0.605 further confirms that there is no significant difference, suggesting that the acute inflammatory response indicated by neutrophil levels is comparable in both *B. pseudomallei* and *B. cepacia infections*.

In summary, the most significant differences were observed in procalcitonin levels, which suggest differing inflammatory responses between the two species. Other parameters, including ESR, CRP, MPV, PDW, PLT, WBC, lymphocyte, and neutrophil counts, did not show significant differences, indicating similar immune responses in infections caused by *B. pseudomallei* and *B. cepacia.*

Sensitivity pattern

B. cepacia

**Figure 6:**
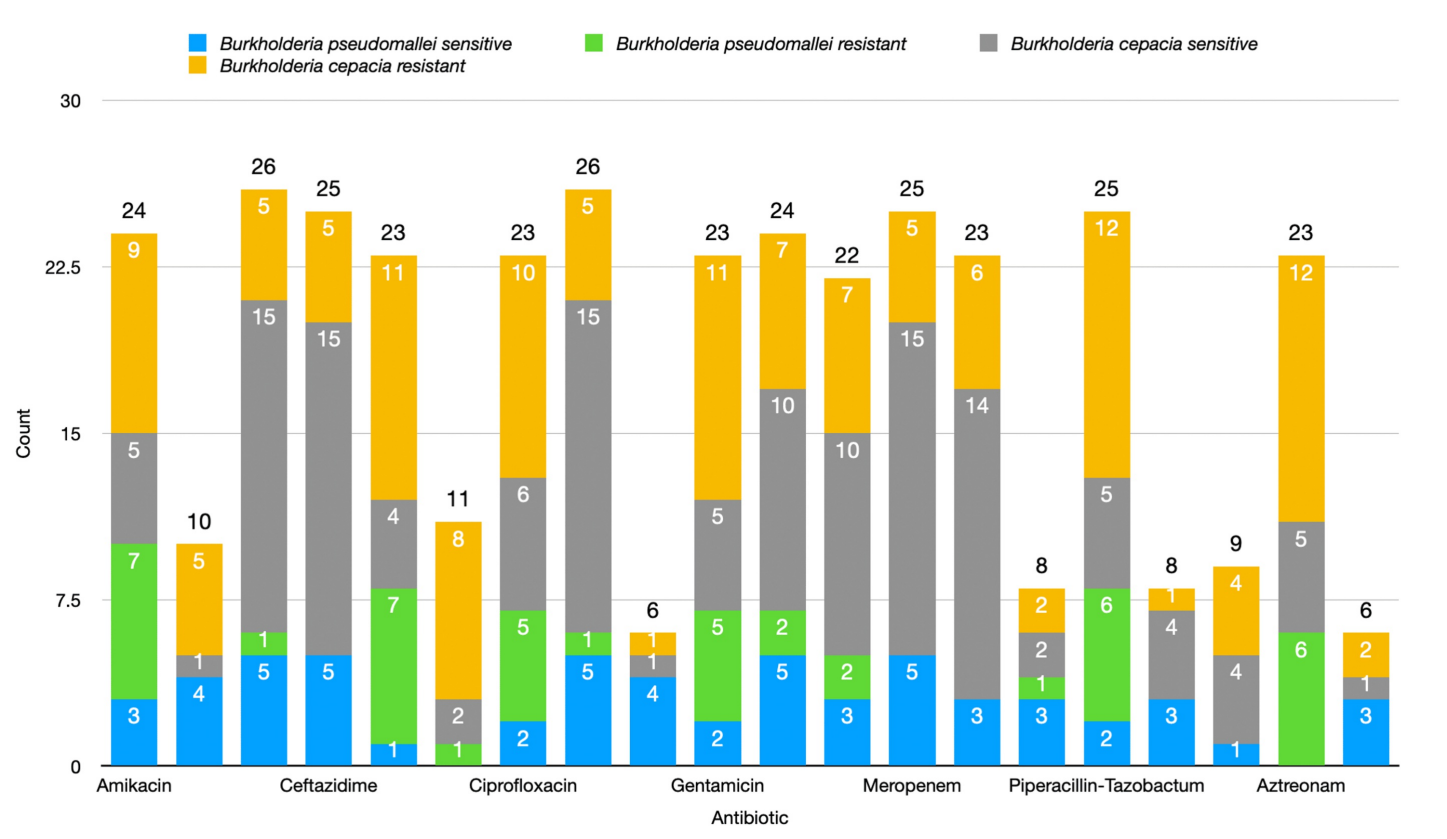
Antibiotic sensitivity pattern of Burkholderia cepacea vs B. pseudomallei

For *B. cepacia*, the data indicate that some antibiotics exhibit high sensitivity as shown in Figure 6. The antibiotics such as cefoperazone/sulbactam, ceftazidime, cotrimoxazole, and meropenem were the most effective, with 15 out of 39 isolates (38.50%) showing sensitivity. Minocycline also performed well, with a sensitivity rate of 14 out of 39 (35.90%). Imipenem and levofloxacin had moderate effectiveness, with 10 sensitive isolates each (25.60%). On the other hand, doxycycline and chloramphenicol showed very low sensitivity, with only 1 out of 39 isolates (2.60%) responding.

In terms of resistance, cefepime and gentamicin demonstrated high resistance, with 11 out of 39 isolates (28.2%) being resistant. Piperacillin/tazobactam and aztreonam were also among the antibiotics with notable resistance, each showing resistance in 12 isolates (30.80%). Ciprofloxacin presented moderate resistance, with 10 out of 39 isolates (25.60%) being resistant. Amoxicillin-clavulanate showed no resistance, while ticarcillin/clavulanic acid exhibited minimal resistance with only 1 out of 39 isolates (2.60%) showing non-susceptibility.

B. pseudomallei

In the case of* B. pseudomallei*, cefoperazone/sulbactam, ceftazidime, imipenem, and meropenem showed the highest sensitivity, with five out of 17 isolates (29.40%) responding positively to these antibiotics. Amoxicillin-clavulanate, cotrimoxazole, doxycycline, levofloxacin, minocycline, ofloxacin, and ticarcillin/clavulanic acid also displayed moderate sensitivity, with three to four isolates (17.60% to 23.50%) being susceptible. Ciprofloxacin, gentamicin, and piperacillin/tazobactam had relatively lower sensitivity, with only two isolates each (11.80%) responding. No sensitivity was recorded for aztreonam and ceftriaxone.

In terms of resistance, amikacin and cefepime showed the highest resistance rates, with seven out of 17 isolates (41.20%) being resistant. Piperacillin/tazobactam and aztreonam were also highly resistant, with six out of 17 isolates (35.30%) displaying non-susceptibility. Ciprofloxacin and gentamicin each had five resistant isolates (29.40%). On the other hand, no resistance was detected for amoxicillin-clavulanate, ceftazidime, doxycycline, meropenem, minocycline, ticarcillin/clavulanic acid, tobramycin, or chloramphenicol.

Key findings

Combining the data from both* B. cepacia* and *B. pseudomallei*, it is evident that some antibiotics, particularly cefoperazone/sulbactam, ceftazidime, and carbapenems such as meropenem and imipenem, show good effectiveness across both bacterial species. However, resistance is notably high for certain antibiotics. In* B. pseudomallei*, amikacin and cefepime exhibited the highest resistance rates, while in *B. cepacia*, piperacillin/tazobactam, aztreonam, and gentamicin were among the most resistant drugs. Ciprofloxacin demonstrated moderate resistance in both species.

These findings suggest that while some broad-spectrum antibiotics such as carbapenems and beta-lactam/beta-lactamase inhibitor combinations remain effective, certain antibiotics (amikacin, cefepime, and aztreonam) may not be as reliable due to significant resistance patterns. This highlights the importance of careful antibiotic selection and susceptibility testing when treating infections caused by these *Burkholderia species*.

## Discussion

This study provides crucial data on* Burkholderia* infections in India, where underreporting and erroneous diagnosis have hindered understanding of their clinical impact. Through a comprehensive clinical and microbiological assessment, we aim to elucidate the demographics, clinical manifestations, and laboratory features of patients infected with *B. cepacia* and *B. pseudomallei*. The analysis of antibiotic susceptibility and resistance patterns will provide insight and ultimately guide the optimization of treatment strategies for these challenging infections.

In our study, male predominance (66.10%) was observed, with most patients between 40 and 60 years of age. This aligns with other studies that have reported higher infection rates in middle-aged male patients, potentially due to occupational or environmental exposure risks associated with these pathogens [7]. Comorbid conditions such as hypertension and diabetes were common, with 35.70% and 39.30% prevalence, respectively. These comorbidities are frequently noted in studies involving *Burkholderia* infections, as they can compromise immune function and increase susceptibility [8].

Microbiological findings and species distribution

In our culture analysis, *B. cepacia* was more frequently isolated, accounting for 67.60% of cases, whereas *B. pseudomallei *accounted for 32.40%. Blood samples yielded the highest number of positive cultures, which aligns with the observations of Gangaram et al. [9], who reported a similar trend of higher isolation rates from blood samples in *Burkholderia* infections. This distribution could reflect the bacteremia-inducing potential of these pathogens, especially in immunocompromised patients [10,11].

Comparison of clinical parameters and laboratory findings

Our study identified elevated procalcitonin levels in patients with *B. pseudomallei*, suggesting a more intense systemic inflammatory response than *B. cepacia* (p = 0.03). This finding is consistent with prior research, indicating that *B. pseudomallei* often provokes a heightened inflammatory response due to its virulence factors [12]. Higher procalcitonin levels in *B. pseudomallei* infections may indicate a stronger inflammatory response, although further analysis of clinical outcomes is needed to confirm its association with infection severity. The elevated CRP levels in patients with *B. pseudomallei* (mean CRP: 197.23 mg/L) approached significance (p = 0.06), also supporting previous reports of stronger inflammatory markers in infections caused by this species [12]. Although we could not find any comparative analysis between the two species, we believe this article will be the first to make such a comparison. Most other laboratory parameters, such as WBC counts and neutrophil levels, showed no significant differences between the two species, a pattern seen in several similar studies [13]. This may indicate comparable acute immune responses, particularly in patients without severe immunosuppression.

Antibiotic sensitivity and resistance patterns

Both *B. cepacia* and *B. pseudomallei* exhibited good sensitivity to certain antibiotics, including cefoperazone/sulbactam, ceftazidime, and meropenem, which aligns with the guidelines suggesting these antibiotics as primary treatment options [14]. However, in the *B. pseudomallei* group, there was a notable resistance to amikacin and cefepime, each demonstrating over 40% resistance, suggesting limited efficacy for these drugs in routine treatment. This is consistent with recent reports highlighting rising resistance to these antibiotics in *Burkholderia* infections [15]. For *B. cepacia*, piperacillin/tazobactam, gentamicin, and aztreonam also showed high resistance rates. These findings are corroborated by studies indicating similar resistance trends, likely due to intrinsic resistance mechanisms and the high adaptability of *Burkholderia* species to antibiotic pressures. Given the resistance to commonly used antibiotics, the use of carbapenems and beta-lactam/beta-lactamase inhibitors appears crucial in managing these infections effectively, as suggested by other authors [16].

Clinical implications and recommendations

These results underscore the importance of species-specific susceptibility testing when treating *Burkholderia *infections. Given the variability in resistance patterns, particularly in multidrug-resistant organisms such as *B. cepacia*, clinicians should exercise caution in antibiotic selection. Furthermore, the significant inflammation markers in *B. pseudomallei* infections could predict prognosis and guide therapeutic decisions. Future research could investigate the genetic basis for these resistance patterns and explore the role of multimodal therapies in managing severe cases, as suggested by recent clinical and diagnostic guidelines [17].

Limitations

This study has several limitations. First, as a single-center study with a relatively small sample size, the findings may not be generalizable to other geographic regions or healthcare settings. Second, the reliance on culture-based identification methods may have underestimated the true prevalence of *Burkholderia* infections, as advanced molecular diagnostics were not employed. Third, while procalcitonin and CRP levels were analyzed, the absence of clinical outcome data limits the ability to correlate inflammatory markers with infection severity or prognosis. Finally, this study did not investigate the genetic mechanisms of antibiotic resistance, which could provide valuable insights into the rising resistance trends observed. Future multicenter studies with larger cohorts, clinical outcome analyses, and genomic approaches are warranted to address these limitations.

## Conclusions

This study offers a unique comparative analysis of *B. cepacia* and *B. pseudomallei*, focusing on clinical, microbiological, and inflammatory responses. By analyzing antibiotic sensitivity and resistance patterns, the findings provide actionable insights for clinicians, particularly in resource-limited settings. Furthermore, identifying elevated procalcitonin levels in*B. pseudomallei* infections adds valuable clinical guidance for diagnosis and treatment. Notably, patients with *B. pseudomallei *infections demonstrated higher procalcitonin levels, suggesting a more pronounced systemic inflammatory response. There is a trend for CRP (approaching significant value) in *B. pseudomallei* group but are only suggestive not meeting statistical significance suggesting the need of more robust clinical trials to seek the CRP as probable diagnostic marker of *B. pseudomallei*. The antibiotic resistance patterns observed underscore the importance of tailored antimicrobial therapy and the necessity for species-specific susceptibility testing to optimize treatment. The study supports the continued use of antibiotics such as cefoperazone/sulbactam and meropenem as effective treatments, while caution is advised when considering agents such as amikacin and cefepime due to noted resistance. Further multicentric, prospective studies involving larger patient cohorts and advanced diagnostic methods are recommended to validate these findings and improve treatment protocols for *Burkholderia* infections.
